# Claudin-Low Breast Cancer; Clinical & Pathological Characteristics

**DOI:** 10.1371/journal.pone.0168669

**Published:** 2017-01-03

**Authors:** Kay Dias, Anna Dvorkin-Gheva, Robin M. Hallett, Ying Wu, John Hassell, Gregory R. Pond, Mark Levine, Tim Whelan, Anita L. Bane

**Affiliations:** 1 Department of Pathology and Molecular Medicine, McMaster University, Hamilton, Ontario, Canada; 2 Department of Oncology, McMaster University, Hamilton, Ontario, Canada; 3 Department of Biochemistry and Biomedical Sciences, McMaster University, Hamilton, Ontario, Canada; Fu Jen Catholic University, TAIWAN

## Abstract

Claudin-low breast cancer is a molecular type of breast cancer originally identified by gene expression profiling and reportedly associated with poor survival. Claudin-low tumors have been recognised to preferentially display a triple-negative phenotype, however only a minority of triple-negative breast cancers are claudin-low. We sought to identify an immunohistochemical profile for claudin-low tumors that could facilitate their identification in formalin fixed paraffin embedded tumor material. First, an *in silico* collection of ~1600 human breast cancer expression profiles was assembled and all claudin-low tumors identified. Second, genes differentially expressed between claudin-low tumors and all other molecular subtypes of breast cancer were identified. Third, a number of these top differentially expressed genes were tested using immunohistochemistry for expression in a diverse panel of breast cancer cell lines to determine their specificity for claudin-low tumors. Finally, the immunohistochemical panel found to be most characteristic of claudin-low tumors was examined in a cohort of 942 formalin fixed paraffin embedded human breast cancers with >10 years clinical follow-up to evaluate the clinico-pathologic and survival characteristics of this tumor subtype. Using this approach we determined that claudin-low breast cancer is typically negative for ER, PR, HER2, claudin 3, claudin 4, claudin 7 and E-cadherin. Claudin-low tumors identified with this immunohistochemical panel, were associated with young age of onset, higher tumor grade, larger tumor size, extensive lymphocytic infiltrate and a circumscribed tumor margin. Patients with claudin-low tumors had a worse overall survival when compared to patients with luminal A type breast cancer. Interestingly, claudin-low tumors were associated with a low local recurrence rate following breast conserving therapy. In conclusion, a limited panel of antibodies can facilitate the identification of claudin-low tumors. Furthermore, claudin-low tumors identified in this manner display similar clinical, pathologic and survival characteristics to claudin-low tumors identified from fresh frozen tumor material using gene expression profiling.

## Introduction

In 2007, while conducting comparative gene expression analysis between transgenic mouse models of breast cancer and human breast cancer data sets, Herschkowitz et al discovered a novel molecular subtype of breast cancer which they named ‘claudin-low’ (CL) [1]. This subtype was characterized by the low expression of genes involved in tight junctions and epithelial cell-cell adhesion, including claudins 3, 4 and 7, occludin and E-cadherin. In addition, the human CL tumors showed low expression of luminal epithelial genes and high expression of lymphocyte and endothelial cell markers [1].

Subsequent to this report, a number of groups have further characterized this new tumor subtype and shown that CL tumors account for 7–14% of all invasive breast cancers, are enriched for genes associated with epithelial to mesenchymal transition (EMT), immune cell infiltration, IFNγ activation, mammary stem cells/breast tumor initiating cells and typically demonstrate high levels of genomic instability [2–5]. Pathologic examination of a limited number of tumors fitting this category, have shown a higher than expected prevalence of medullary-like and metaplastic special-type tumors and tumors with a triple negative (TN) phenotype. Clinically, they have been associated with a poor prognosis with some evidence that they may be relatively resistant to conventional chemotherapeutic agents [2, 3].

These studies have all identified the CL subtype by means of gene expression profiling, however this technique requires fresh frozen tumor material, which is not available for the majority of breast cancer patients. We sought to determine an immunohistochemical (IHC) profile that could identify CL tumors in formalin fixed paraffin embedded (FFPE) tumor specimens. Such a profile would enable us and others, to examine the pathologic and clinical significance of the CL tumors in larger cohorts of archived FFPE tumor specimens providing a more comprehensive analysis of the tumor subtype.

To this end we collated an *in silico* data base comprising the expression profiles of approximately 1600 individual breast cancers. Tumors comprising this database were classified into the known molecular subtypes; luminal A, luminal B, HER-2 enriched, basal-like, normal-like, molecular apocrine and CL using previously published classifiers [2, 6–8]. Genes differentially expressed between the CL and all other molecular subtypes of breast cancer were identified. The protein products of some of the top differentially expressed genes were examined using IHC for their ability to identify the CL subtype in a diverse panel of breast cancer cell lines. Finally, the panel of IHC markers that in combination optimally discriminated between CL tumor cell lines and cell lines of other molecular subtypes was examined in a large tissue microarray (TMA) of primary invasive human breast cancers. The tumors identified in this TMA cohort as CL using the surrogate IHC profile described were compared with tumors of other molecular subtypes for clinical-pathologic characteristics of known prognostic importance, disease free survival (DFS), overall survival (OS) and local recurrence rates (LRR) (S1 Fig).

## Materials and Methods

### *In silico* data collection

In the course of our study we analyzed the gene expression profiles *in silico* of 7 external datasets, obtained using Affymetrix HG-U133A GeneChip arrays. These profiles were deposited in the Gene Expression Omibus (GEO) (accession numbers of the datasets are: GSE3494, GSE1456, GSE7390, GSE2034, GSE6532, GSE17705 and GSE25066) and comprise a total of 2,027 samples (S1 Table). Redundant samples were removed as previously described reducing the number of unique samples to 1,695 [8]. All samples used for our study were normalized with frozen Robust Multi-array Analysis (fRMA) [9], technical variation was removed using the ComBat and DWD (Distance-Weighted Discrimination) methods [10, 11]. After combining all datasets Spearman correlation coefficients for pair-wise comparisons of samples using 68 house-keeping probe sets were computed, and only samples exhibiting a correlation higher than 0.95 with at least half of the dataset were selected for further classification. The latter filtering method yielded a dataset comprising 1,593 human breast tumor sample transcript profiles.

### *In silico* molecular subtype assignment

The 1,593 tumors were assigned to one of 7 molecular subtypes (luminal A, luminal B, HER-2-enriched, basal-like, normal-like, molecular apocrine or CL) using 710 genes obtained from previously published gene classifiers [6–8, 12]. In brief the standardized centroid was computed for each subtype by taking the average expression of each gene across the subtype and dividing it by the standard deviation of expression of that gene across that subtype. Spearman rank correlation coefficient was computed for each sample relative to each of the 7 reference centroids, and the subtype was assigned based on the highest correlation coefficient. For the assignment we used a coefficient cut-off of 0.3; therefore 1,196 samples were classified into the 7 established molecular subtypes as previously described [8]. This classification yielded 80 (6.69%) samples defined as CL.

### Identification of genes differentially expressed between CL tumors and all other molecular subtypes

To identify gene patterns unique to CL tumors we used the “limma” package (Bioconductor; [13]) to compare the expression profiles of samples assigned to the CL subtype to those of the all other subtypes (6 pair-wise comparisons in total). To this end the moderated F-statistic was used, followed by Benjamini-Yekutieli adjustment for multiple testing [14]. The 710 genes belonging to the molecular subtype classifier were used for this analysis and only genes differentially expressed with at least a 2 fold change were examined further.

### Breast tumor cell lines representative of the molecular subtypes of breast cancer

A total of 9 breast cancer cell lines known to replicate the luminal, basal and CL subtypes of primary human breast tumor samples were grown using the recommended culture conditions [15], these included 5 luminal (MCF7, ZR751, SKBR3, BT474, MDA-MB-361), 2 basal-like (BT20, HCC 1954) and 2 CL cell lines (BT549, MDA-MB-231) [15]. Cells were fixed and paraffin embedded as detailed in the supplementary information (S1 File). The paraffin embedded cell lines were stained immunohistochemically for ER, PR, HER2, CK5, EGFR, E-cadherin, claudin 3, claudin 4, claudin 7 and CD24 using methods as listed in S2 Table.

### Human FFPE breast tumor material

942 T1 or T2, node negative breast cancers treated with breast conserving therapy, which had been accrued as part of the Accelerated Hypofractionated Whole Breast Irradiation (AHWBI) trial (see S1 File) were available for analysis [16, 17]. This cohort had 10 years of clinical follow-up available including, LR, DFS and OS.

A single hematoxylin and eosin (H&E) stained section, representative of each invasive carcinoma was reviewed by the study pathologist (ALB). Tumors were assessed for tumor type, grade, lympho-vascular space invasion (LVI), extensive lymphocytic infiltrate and margin circumscription. Tumors were classified according to the World Health Organization (WHO) histologic classification of breast tumors [18] and graded using the Nottingham grading system [19]. The lymphocytic tumor infiltrate was graded on a four point scale; none, minimal, moderate and extensive (see S1 File). The study pathologist was blinded to the patient outcome during the review process.

The invasive tumor component of each H&E stained section was encircled with permanent ink for TMA construction. Three 0.6mm cores of tissue were taken from the paraffin tumor block and used for TMA construction (Pathology Device, Sun Praire, WI) as previously described [16].

Four μm sections were cut from all TMAs and immunohistochemical staining for ER, PR, HER2, CK5, EGFR, Ki67, claudin 3, claudin 4, claudin 7, E-cadherin, CD24, CD44, ALDH1 and CD8 was performed using methods as listed in S2 Table. Microwave antigen retrieval was carried out in a Micromed T/T Mega Microwave Processing Lab Station (ESBE Scientific, Markham, Ontario, Canada). Sections were developed with diaminobenzidinetetrahydrochloride (DAB) and counterstained in Mayer’s hematoxylin.

Each of the immunohistochemical TMA and tumor cell line stained sections was scored using Allred’s scoring method [20], which adds scores for the intensity of staining (absent: 0, weak: 1, moderate: 2, and strong: 3) to the percentage of cells stained (none: 0, <1%: 1, 1–10%: 2, 11–33%: 3, 34–66%: 4 and 67–100%: 5) to yield a ‘raw’ score of 0 or 2–8. Previously validated cut-offs for ER and PR were used (0, 2 = negative, 3–8 = positive) [21, 22]. Strong complete membranous staining was assessed for HER2 and the cut-off of ≥ 6 was used to indicate positivity [23]. For CK5, EGFR, CD24, CD44 and ALDH1 a score of ≥ 4, was considered positive. For claudin 3, 4, 7 and E-cadherin a score of ≤4 was considered ‘low’ expression. For Ki67 a minimum of 100 tumor nuclei were counted per core and the tumor was considered Ki67 ‘low’ if the percentage of positively stained nuclei was <14% and Ki67 ‘high’ is the percentage of positively stained nuclei was ≥14% [12, 24]. The raw score data from the TMAs were reformatted using a TMA deconvoluter software program into a format suitable for statistical analysis [25]. The highest score from each TMA tumor triplicate was entered into the statistical analysis.

Tumors that had an Allred score of 4 or 5 for HER2 were considered equivocal or indeterminate for HER2 overexpression and fluorescent *in situ* hybridization (FISH) was performed on representative tumor sections using the HER2 DNA probe kit (Path-Vysion, Vysis) as previously described [16]. A HER2 to centromere 17 ratio of ≥2 was considered to indicate amplification in accordance with guidelines [23].

Tumors were classified as luminal A if they expressed ER or PR and were negative for HER2 and were Ki67 ‘low’; luminal B if they expressed ER or PR and were either HER2 positive or were Ki67 ‘high’; HER2 enriched if they did not express ER or PR but were positive for HER2; basal-like if they did not express ER, PR or HER2 (triple negative) but expressed CK5 and/or EGFR and CL if they did not express ER or PR or HER2 (TN) and had low expression of at least two of the following markers E-cadherin, claudin 3, claudin 4 and claudin 7 [16, 26]. Tumors were considered unclassified for molecular subtype when results of one or more IHC markers were unavailable due to loss of invasive tumor on sequential TMA slides.

### Ethics statement

The AHWBI FFPE samples were obtained with research ethics board (REB) approval. We did not pursue individual patient consent for tumor samples and this was not required by our REB process for a number of reasons, including. 1. The trial was performed many years ago from 1993 to 1996. As such we recognized that many patients had died and many others were likely to have changed residence. 2. We believed that contacting patients' families would be difficult and likely upsetting given the limited study we were conducting. 3. The analysis we planned is limited to IHC testing and while this is linked to the patients' original data base from the trial all the data was grouped and made anonymous.

### Statistical analysis

Summary statistics were used to describe the patient cohort and outcomes. The Kaplan-Meier method was used to estimate time-to-event outcomes. Comparison between different subtypes was performed using the log-rank tests, χ^2^ test, Cochran-Armitage test for trend or Kruskal-Wallis test as appropriate. All statistical tests were two-sided and statistical significance was defined as a p-value of 0.05 or less. Statistical analyses were performed in SAS version 9.0 (SAS Institute, Cary, NC) and figures were plotted using R version 3.2.2 (www.r-project.org).

## Results

### Identification of CL subtype using gene expression profiling

We compiled gene expression profiles from 7 independent datasets for which clinical follow-up was available. Together these data sets represent 1,593 non-redundant tumors. Using established subtype classifiers [2, 6–8] 1,196 (75.1%) out of the 1,593 samples were classified into one of the seven molecular subtypes; luminal A (n = 340, 28.43%), luminal B (n = 216, 18.06%), HER2-enriched (n = 81, 6.77%), basal (n = 280, 23.41%), normal-like (n = 179, 14.97%), molecular apocrine (n = 20, 1.68%) and CL (n = 79, 8.4%). The OS and DFS of the molecular subtypes are presented in S2 Fig.

### Identification of genes differentially expressed between the CL subtype and all other molecular subtypes of breast cancer

**Fig 1:**
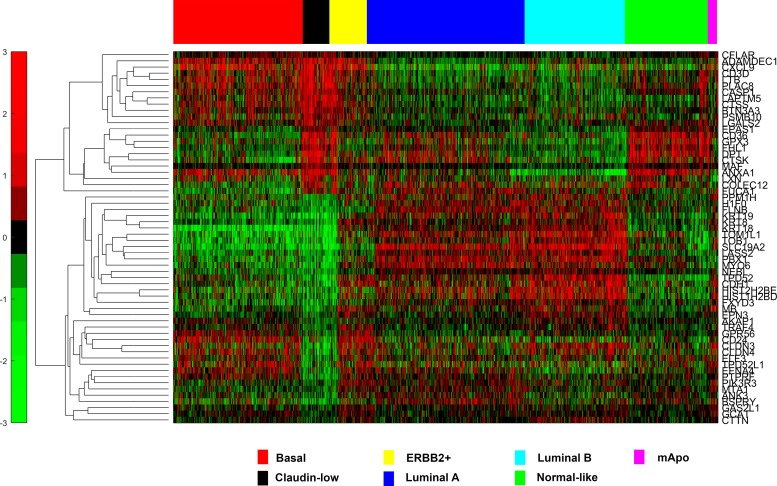
Hierarchical Clustering of 1,593 breast tumor samples using the 60 genes identified as differentially expressed between CL tumors and tumors of all other molecular subtypes

Using a pair wise comparison 60 genes were identified as being differentially expressed by CL tumors relative to all other molecular subtypes of breast cancer (Table 1, Fig 1). Some of the genes we found to be expressed at significantly lower levels in the CL subtype relative to all other subtypes included E-cadherin, claudin 3, claudin 4 and genes associated with luminal epithelial differentiation including CD24, CK8 and CK18 as previously described [1, 2, 5]. As other authors have identified low expression of claudin 7 as being a characteristic feature of CL tumors, we examined the expression of claudin 7 across the breast cancer subtypes in our *in silico* database. We found that claudin 7 was expressed at a lower level in CL tumors relative to all other subtypes with the exception of basal-like tumors (S3 Fig)

**Table 1:** Genes differentially expressed between CL tumors and all other molecular subtypes of breast cancer using gene-expression profiling.

Gene Symbol	Gene Name
**Genes up-regulated in CL tumors vs. all other molecular subtypes of breast cancer**	
ADAMDEC1	ADAM-like, decysin 1
ANXA1	annexin A1
BTN3A3	butyrophilin, subfamily 3, member A3
CASP1	caspase 1, apoptosis-related cysteine peptidase (interleukin 1, beta, convertase)
CD36	CD36 molecule (thrombospondin receptor)
CD3D	CD3d molecule, delta (CD3-TCR complex)
CFLAR	CASP8 and FADD-like apoptosis regulator
COLEC12	collectin sub-family member 12
CTSK	cathepsin K
CTSS	cathepsin S
CXCL9	chemokine (C-X-C motif) ligand 9
DPT	Dermatopontin
EPAS1	endothelial PAS domain protein 1
FHL1	four and a half LIM domains 1
FUCA1	fucosidase, alpha-L- 1, tissue
GPX3	glutathione peroxidase 3 (plasma)
LAPTM5	lysosomal protein transmembrane 5
LGALS2	lectin, galactoside-binding, soluble, 2
LTB	lymphotoxin beta (TNF superfamily, member 3)
LXN	Latexin
MAF	v-maf musculoaponeurotic fibrosarcoma oncogene homolog (avian)
PLAC8	placenta-specific 8
PSMB10	proteasome (prosome, macropain) subunit, beta type, 10
**Genes Down-regulated in CL tumors vs. all other molecular subtypes of breast cancer**	
AKAP1	A kinase (PRKA) anchor protein 1
ANK3	ankyrin 3, node of Ranvier (ankyrin G)
BSPRY	B-box and SPRY domain containing
CD24	CD24 molecule
CDH1	cadherin 1, type 1, E-cadherin (epithelial)
CLDN3	claudin 3
CLDN4	claudin 4
CTTN	Cortactin
EFNA4	ephrin-A4
ELF3	E74-like factor 3 (ets domain transcription factor, epithelial-specific)
EPN3	epsin 3
FLNB	filamin B, beta
FXYD3	FXYD domain containing ion transport regulator 3
GAS2L1	growth arrest-specific 2 like 1
GCAT	glycine C-acetyltransferase
GPR56	G protein-coupled receptor 56
H1F0	H1 histone family, member 0
HIST1H2BD	histone cluster 1, H2bd
HIST2H2BE	histone cluster 2, H2be
KRT18	keratin 18
KRT19	keratin 19
KRT8	keratin 8
LASS2	LAG1 homolog, ceramide synthase 2
MB	Myoglobin
MTA1	metastasis associated 1
MYO6	myosin VI
NEBL	Nebulette
PBX1	pre-B-cell leukemia homeobox 1
PIK3R3	phosphoinositide-3-kinase, regulatory subunit 3 (gamma)
PPM1H	protein phosphatase, Mg2+/Mn2+ dependent, 1H
PTPRF	protein tyrosine phosphatase, receptor type, F
SLC19A2	solute carrier family 19 (thiamine transporter), member 2
TOB1	transducer of ERBB2, 1
TOM1L1	target of myb1 (chicken)-like 1
TPD52	tumor protein D52
TPD52L1	tumor protein D52-like 1
TRAF4	TNF receptor-associated factor 4

Genes expressed at significantly higher levels in CL tumors relative to other molecular subtypes included many genes involved in immune response, host defence and apoptosis including ADAMDEC1, BTN3A3, CD3D, COLEC12, CXCL9, LTB, PSMB10 and MAF (Table 1). The preponderance of immune related genes in CL tumors is thought to reflect considerable immune cells infiltrate in these tumors [5, 27].

Genes associated with tumor invasiveness and epithelial to mesenchymal transition (EMT); CTSK and PLAC8 respectively (Table 1), were also identified as being upregulated in CL tumors [28]. An enrichment for gene signatures associated with EMT has previously been demonstrated for CL tumors [4, 29–31].

### Evaluation of immunohistochemical markers of CL tumors in breast cancer cell lines

We next sought to capture the CL phenotype identified *in silico* with a simple IHC based assay. We first compiled a panel of breast cancer cell lines of known molecular subtype including 2 CL cell lines; these were fixed in formalin and embedded in paraffin blocks to ensure that they were processed in a manner analagous to human tumor samples and profiled for ER, PR, HER2, CK5/6 and EGFR as previously described [16]. Taking into consideration the availability of high quality antibodies and our *in silico* results (Table 1) five antibodies were selected for testing; E-cadherin, claudin 3, claudin 4, claudin 7 and CD24. All of these 5 markers had been shown *in silico* to be differentially expressed in CL tumors relative to other molecular subtypes of breast cancer.

As illustrated in Table 2, those cell lines known to be CL had absent or low expression of the luminal epithelial markers (ER, PR, HER-2) and the epithelial cell-cell adhesion markers (claudin 3, claudin 4, claudin 7 and E-cadherin) at the cut points described. In contrast, the luminal cell lines were positive for at least one of the luminal epithelial cell markers (ER, PR or HER2) together with at least two of the epithelial cell-cell adhesion markers (claudin 3, claudin 4, claudin 7 and E-cadherin). The basal cell lines were negative for all luminal epithelial cell markers (ER, PR & HER2), positive for myoepithelial cell markers (CK5/6 & EGFR) and positive for at least 3 of the epithelial cell-cell adhesion proteins (claudin 3, claudin 4, claudin7 and E-cadherin). None of the profiled cell lines expressed CD24, which may possibly be an artifact of cell culture.

**Table 2:** Expression of Immunohistochemical Markers in Breast Cancer Cell Lines.

	Molecular Subtype	ER	PR	HER-2	CK5/6	EGFR	Claudin 3	Claudin 4	Claudin 7	E-Cadherin	CD24
MCF 7	Luminal	7	5	0	0	0	7	7	8	6	0
ZR751	Luminal	7	4	5	0	0	3	3	7	8	0
SKBR3	Luminal (HER-2 amp)	0	0	8	0	6	3	6	7	0	0
BT474	Luminal (HER-2 amp)	7	4	8	0	4	8	8	8	6	0
MDAMB361	Luminal (HER-2 amp)	4	0	8	0	4	8	7	8	7	0
BT20	Basal	0	0	0	7	8	0	5	7	6	0
HCC1954	Basal (HER2-amp)	0	0	8	8	8	0	8	8	8	0
BT549	Claudin-low	0	0	0	0	7	0	0	0	0	0
MDAMB231	Claudin-low	0	0	0	0	8	0	3	0	0	0

From these experiments we concluded that a surrogate IHC panel for the identification of tumors belonging to the CL molecular subtype would be TN (ER-, PR- and HER2-), together with low or absent expression of at least 2 of the 4 epithelial cell-cell adhesion proteins, claudin 3, claudin 4, claudin 7 and E-cadherin.

### Clinical-pathologic characteristics of CL tumors using TMAs of human breast cancers

We next sought to examine the clinical-pathologic tumor features and survival characteristics of CL tumors identified using the surrogate IHC panel in a large cohort of invasive breast cancer with long-term clinical outcome data.

**Fig 2:**
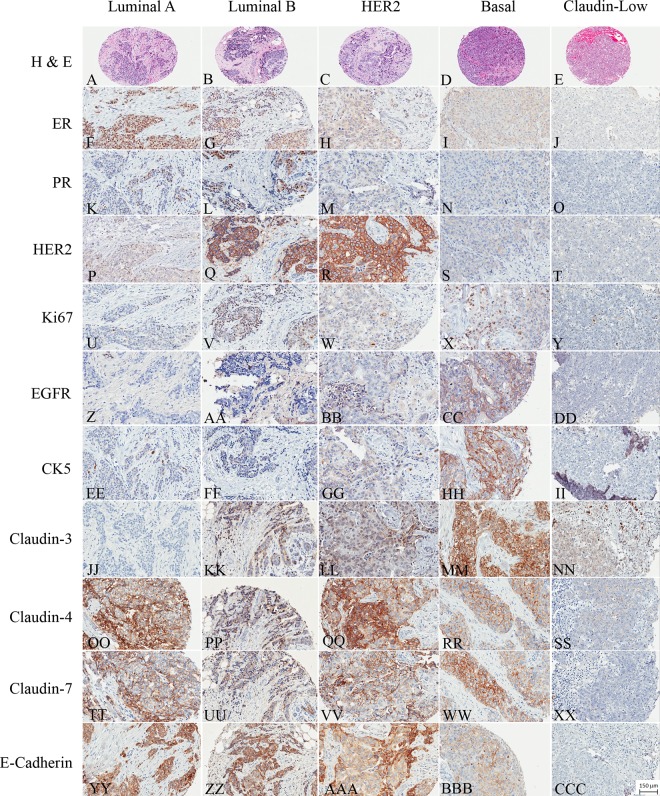
Representative H&E stained image of luminal A [A], luminal B [B], HER2 [C], basal [D] and Claudin-low [E] tumors. ER staining in luminal A [F], luminal B [G], HER2 [H], basal [I] and Claudin-low [J] tumors. PR staining in luminal A [K], luminal B [L], HER2 [M], basal [N] and Claudin-low [O] tumors. HER2 staining in luminal A [P], luminal B [Q], HER2 [R], basal [S] and Claudin-low [T] tumors. Ki67 staining in luminal A [U], luminal B [V], HER2 [W], basal [X] and Claudin-low [Y] tumors. EGFR staining in luminal A [Z], luminal B [AA], HER2 [BB], basal [CC] and Claudin-low [DD] tumors. CK5 staining in luminal A [EE], luminal B [FF], HER2 [GG], basal [HH] and Claudin-low [II] tumors. Claudin-3 staining in luminal A [JJ], luminal B [KK], HER2 [LL], basal [MM] and Claudin-low [NN] tumors. Claudin-4 staining in luminal A [OO], luminal B [PP], HER2 [QQ], basal [RR] and Claudin-low [SS] tumors. Claudin-7 staining in luminal A [TT], luminal B [UU], HER2 [VV], basal [WW] and Claudin-low [XX] tumors. E-cadherin staining in luminal A [YY], luminal B [ZZ], HER2 [AAA], basal [BBB] and Claudin-low [CCC] tumors.

942 primary invasive breast tumors arrayed in triplicate in TMAs were examined for the expression of a panel of IHC markers; ER, PR, HER-2, Ki67, CK5/6, EGFR, claudin 3, claudin 4, claudin 7 and E-cadherin to approximate the known molecular subtypes of breast cancer [16, 26]. 776 (82.4%) of the 942 tumors could be classified into one of the five molecular subtypes; luminal A (n = 389, 41.3%), luminal B (n = 234, 24.8%), HER2 enriched (n = 21, 2.2%), basal-like (n = 53, 5.6%) and CL (n = 79, 8.4%) as described in the material and methods (Fig 2). 166 (17.6%) cases could not be classified into one of the molecular subtypes due to unavailable IHC data and were placed into an ‘unclassified’ category for purposes of analysis. A suitable surrogate IHC profile for the molecular apocrine group or the normal-like group is not available and these subtypes were not considered further in this data set.

The clinical-pathologic features are listed in Table 3. There were significant differences in median patient age, tumor size, grade, extensive lymphocytic infiltrate and margin circumscription between subtypes. Patients also varied according to adjuvant systemic therapy received.

**Table 3:** Clinical-pathologic Characteristics of Invasive Breast Cancers According to Molecular Subtypes in the TMA cohort.

		Claudin-Low	Luminal A	Luminal B	HER2-E	Basal	Not Classifiable	p-value*	Non Claudin-Low	p-value^&^
**N (%)**		79 (8.4%)	389 (41.3)	234 (24.8)	21 (2.2)	53 (5.6)	166 (17.6)		863	
**Age**	**Mean (sd)**	52.8 (11.9)	60.5 (10.3)	58.3 (11.8)	56.4 (11.1)	51.9 (10.9)	59.0 (11.1)	<0.001	59.0 (11.1)	<0.001
	**≥50**	47 (59.5)	317 (81.5)	161 (68.8)	14 (66.7)	29 (54.7)	130 (78.3)	<0.001	651 (75.4)	0.003
**Tumor Grade**	**I**	5/72 (6.9)	109/360 (30.3)	26/209 (12.4)	1/19 (5.3)	1 (2.3)	36 (24.3)	<0.001	173/779 (22.2)	
	**II**	18 (25.0)	238 (66.1)	143 (68.4)	10 (52.6)	6 (14.0)	91 (61.5)		488 (62.6)	<0.001
	**III**	49 (68.1)	13 (3.6)	40 (19.1)	8 (42.1)	36 (83.7)	21 (14.2)		118 (15.2)	
**Tumor Size**	**≥2 cm**	26 (32.9)	51 (13.1)	61 (26.1)	7 (33.3)	18 (34.0)	23 (13.9)	<0.001	160 (18.5)	0.005
**Prior Treatment**	**Tamoxifen**	16 (20.3)	199 (51.2)	111 (47.4)	2 (9.5)	5 (9.4)	64 (38.6)	<0.001	381 (44.2)	<0.001
	**Chemotherapy**	32 (40.5)	11 (2.8)	18 (7.7)	9 (42.9)	31 (58.5)	15 (9.0)		84 (9.7)	
	**None**	31 (39.2)	179 (46.0)	105 (44.9)	10 (47.6)	17 (32.1)	87 (52.4)		398 (46.1)	
**Lymphocytic Infiltrate**	**Extensive**	31/74 (41.9)	4/377 (1.1)	20/219 (9.1)	7/21 (33.3)	22/48 (45.8)	12/156 (7.7)	<0.001		<0.001
									65/821 (7.9)	
**Circumscribed Margins**	**Positive**	35/79 (44.3)	66/389 (17.0)	53/233 (22.8)	6/21 (28.6)	20/52 (38.5)	34/166 (20.5)	<0.001	179/861 (20.8)	
										<0.001
**LVI**	**Positive**	10/79 (12.7)	42/389 (10.8)	49/233 (21.0)	1/21 (4.8)	9/52 (17.3)	13/166 (7.8)	<0.001	114/861 (13.2)	1
**ALDH1**	**Positive**	12/70 (17.1)	14/321 (4.4)	16/217 (7.4)	2/19 (10.5)	11/42 (26.2)	4/48 (8.3)	<0.001	47/647 (7.3)	0.01
**CD44**^**+**^ **/CD24**^**-/low**^	**N (%)**	21/67 (31.3)	68/308 (22.1)	39/215 (18.1)	1/18 (5.6)	17/42 (40.5)	14/59 (23.7)	0.006	139/642 (21.7)	0.09
**Overall Survival**	**N (%) Deaths**	18 (22.8)	75 (19.3)	50 (21.4)	6 (28.6)	15 (28.3)	40 (24.1)	0.4	186 (21.5)	0.68
	**3-year OS**	93.6 (85.3–97.3)	98.2 (96.3–99.1)	97.9 (94.9–99.4)	90.5 (67.0–97.5)	84.6 (71.6–92.0)	94.5 (89.7–97.1)		96.4 (94.9–97.4)	
	**5-year OS**	89.7 (80.4–94.7)	95.9 (93.3–97.4)	93.5 (89.4–96.0)	90.5 (67.0–97.5)	82.6 (69.3–90.6)	92.0 (86.6–95.3)		93.6 (91.7–95.0)	
	**10-year OS**	81.6 (70.9–88.7)	85.8 (81.8–89.0)	83.9 (78.4–88.2)	90.5 (67.0–97.5)	78.6 (64.7–87.6)	81.0 (74.0–86.3)		84.1 (81.4–86.4)	
**Local Recurrence**	**N (%) Recurrences**	1 (1.3)	21 (5.4)	16 (6.8)	6 (28.6)	4 (7.5)	13 (7.8)	<0.001	60 (6.9)	0.068
	**5-year LR**	98.7 (91.2–99.8)	99.0 (97.2–99.6)	96.4 (92.9–98.2)	84.2 (58.7–94.6)	93.9 (82.3–98.0)	96.7 (92.3–98.6)		97.2 (95.8–98.1)	
	**10-year LR**	98.7 (91.2–99.8)	95.0 (92.0–96.8)	93.1 (88.6–95.9)	78.9 (53.2–91.5)	91.4 (78.5–96.7)	92.3 (86.4–95.7)		93.4 (91.3–94.9)	
**Disease-Free Survival**	**N (%) Events**	27 (34.2)	126 (32.4)	90 (38.5)	15 (71.4)	21 (39.6)	69 (41.6)	0.002	321 (37.2)	0.71
	**5-year DFS**	79.3 (68.4–86.8)	86.6 (82.8–89.6)	81.0 (75.3–85.5)	71.4 (47.2–86.0)	73.1 (58.8–83.1)	77.9 (70.8–83.6)		82.2 (79.5–84.6)	
	**10-year DFS**	72.5 (60.9–81.1)	72.2 (67.4–76.4)	65.7 (59.1–71.5)	56.7 (33.3–74.7)	61.2 (46.6–73.0)	64.4 (56.4–71.3)		67.9 (64.6–71.0)	

* = p-value for comparison between the 6 different subtypes

^&^ = p-value for comparing claudin-low versus non-claudin low

**Fig 3:**
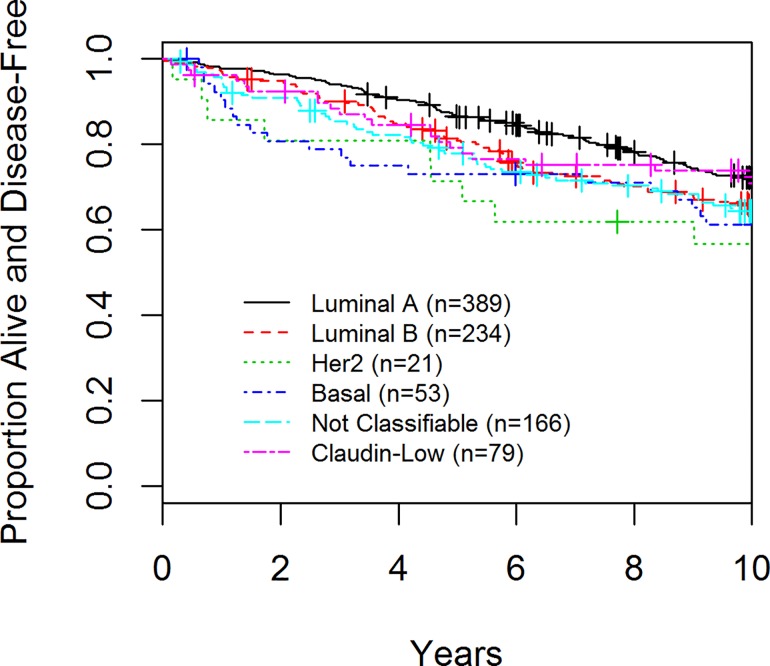
Disease-Free Survival (DFS) of Patients enrolled in the AHWBI trial by Tumor Subtype

When compared to all other subtypes combined, CL tumors were more likely to occur at a younger age (mean = 52.8 versus 59.0, p<0.001) be high grade (68.1% versus 15.2% were grade III, p<0.001), larger size (32.9% versus 18.5% were ≥2cm, p = 0.005) and to be characterized by an extensive lymphocytic infiltrate (41.9% versus 7.9%, p<0.001) and to have pushing or circumscribed tumor margins (44.3% versus 20.8%, p<0.001) (Table 3, Fig 3).

### CL tumors and association with markers of breast cancer stem cells/tumor initiating cells

We examined the association between CL tumors in our TMA cohort and known markers of breast cancer stem cells/tumor initiating cells including ALDH1 and CD44^hi^/CD24^-/low^ [32–35]. When compared to all other subtypes combined, CL tumors were more likely to express ALDH1 (17.1% versus 7.3%, p = 0.010) and they showed a trend towards an association with the CD44^hi^/CD24^-/low^ phenotype (31.3% versus 21.7%, p = 0.090) (Table 3, S4 Fig).

### Prognosis

**Fig 4:**
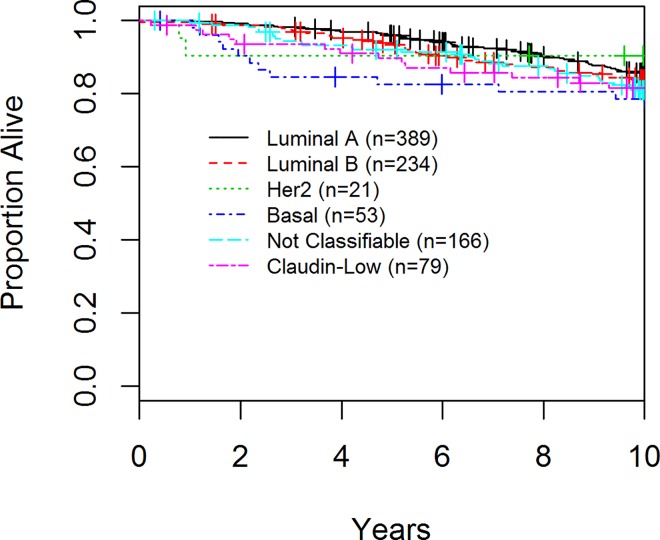
Overall Survival (OS) of Patients enrolled in the AHWBI trial by Tumor Subtype

**Fig 5:**
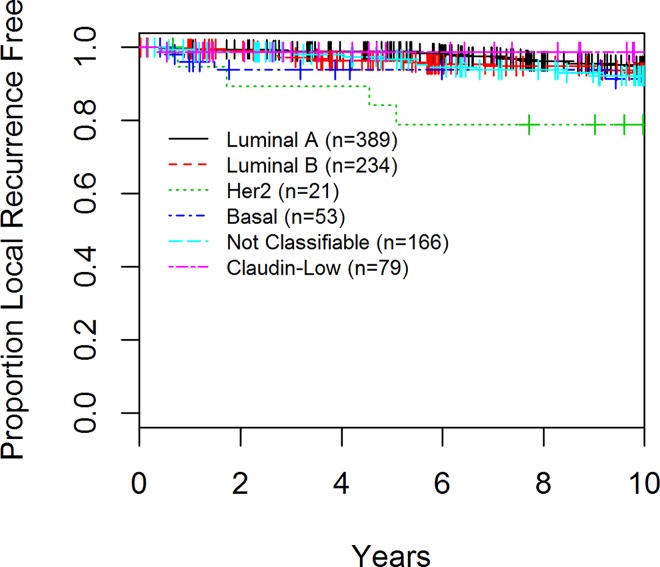
Local Recurrence (LR) of Patients enrolled in the AHWBI trial by Tumor Subtype

In the final cohort of 942 patients, 348 had a disease-related event, 61 had a local recurrence and 204 deaths were observed. With respect to DFS, luminal A and CL cancers had the best prognosis at 10 years (72.2% and 72.5% respectively) whereas basal-like and HER2 enriched had the worst (61.2 and 56.7% respectively), (p = 0.002; Table 3, Fig 3). The majority of the recurrences associated with CL tumors occurred within the first 5 years of diagnosis. Although not statistically significant (p = 0.40) the groups with the worst OS were patients with basal-like and CL tumors (Table 3, Fig 4). With regards to local recurrence (LR), only 1 of 79 (1.3%) patients with a CL tumor had a LR as compared to 21 (5.4%) of luminal A, 15 (6.8%) of luminal B, 6 (28.6%) of the HER2 enriched patients, 4 (7.5%) of the basal-like (p<0.001, Table 3, Fig 5).

## Discussion

An appropriate IHC surrogate approach for the classification of CL breast tumors has not been heretofore rigorously identified limiting our ability to recognize and study this subtype in further detail. To this end, using an *in silico* dataset of 1,196 breast tumors each of which could be assigned to one of 7 molecular subtypes; luminal A, luminal B, HER2 enriched, basal-like, normal-like, molecular apocrine and CL we identified that 6.69% (n = 80) of the tumors were CL. These CL tumors differed from other molecular subtypes by their lower expression of epithelial cell-cell adhesion factors, markers of luminal epithelial cell differentiation and by their elevated expression of genes involved in immunity and host defence and tumor cell invasiveness and EMT. These results reaffirm the dominant biological pathways functioning in CL tumors. Breast cancer cell lines reflective of the CL subtype were also found to express low or absent levels of the epithelial cell-cell adhesion proteins examined (E-cadherin, claudin 3, claudin 4 and claudin 7) and markers of breast luminal epithelial cell differentiation (ER, PR and HER-2). This unique IHC profile (TN and cell adhesion protein low/absent) could distinguish the CL subtype in cell line preparations from the other breast cancer molecular subtypes studied.

Furthermore, when we examined a large retrospective collection of 942 primary human breast cancers with clinical, pathologic and outcome data for the surrogate CL IHC profile we identified a tumor group with distinguishing morphologic and outcome characteristics. We observed that the incidence of the CL subtype was 8.4% similar to the 7–14% incidence reported previously [2, 5]. Phenotypic characteristics of this subtype when compared to other subtypes included an association with high tumor grade, large tumor size, an extensive lymphocytic infiltrate and circumscribed/pushing tumor margins. Many of these features are component features of medullary or atypical medullary breast cancer, a subtype of TN breast cancer. An association between these medullary-like features and CL tumors has been previously identified [2]. The extensive lymphocytic infiltrate associated with CL tumors in our primary breast tumor cohort correlates well with the preponderance of immune associated genes upregulated in this subtype as identified by our *in silico* experiments.

CL tumors have previously been shown to be enriched for genes associated with mammary stem cells/breast cancer tumor initiating cells, from which it has been inferred that this tumor subtype may be enriched for these primitive cells types and even potentially derived from the malignant transformation of a mammary epithelial stem cell [29, 36–40]. To test if our IHC profile accurately captures this aspect of CL tumor biology we examined the expression of known cancer stem cell/breast tumor initiating cell markers; ALDH1 and CD44^hi^/CD24^low/-^ in our TMA cohort of human tumors [33–35]. The CL subtype was significantly more likely to express the mammary stem cell/breast tumor initiating cell marker ALDH1 (p = 0.01) than non-CL tumors and there was a trend for association between CL tumors and the CD44^hi^/CD24^low/-^ phenotype (p = 0.09). These results suggest that the surrogate IHC profile used is accurately identifying the CL subtype of tumors.

CL tumors as identified by gene expression profiling have been shown to have an outcome intermediate between that of luminal A and poor prognostic subtypes such as luminal B, basal-like and HER2 enriched subtypes [2, 5]. Using the IHC definition described we show that while not statistically significant (p = 0.04) patients with CL had a worse OS at 10 years (81.6%) compared to patients with luminal A disease (85.8%) (Table 3). The relatively good outcome described for patients with CL tumors in our cohort may be attributable to the presence of an extensive lymphocytic infiltrate in many of these tumors. Increased quantities of tumor infiltrating lymphocytes have been shown by a number of investigators to be associated with good outcome in breast cancer in general and TN tumors in particular [41–44]. The relative good outcome for CL tumors in our cohort could also be ascribed to the low tumor stage of all patients eligible for entry onto this trial (T1 /T2 and N0).

This is the first study to report on the LR rates for CL tumors. The CL subtype had the lowest rate of LR (1.3% at 5 and 10 years) of any molecular subtype studied. Given that all patients in this cohort were treated with breast conserving surgery and whole breast irradiation this finding may suggest that CL tumors are particularly sensitive to radiation. However, given the low number of CL tumors and the overall low LR rate in the study population this result should be considered hypothesis generating and would need to be validated in other data sets.

In summary, we have taken a two step approach to identify a surrogate IHC profile to identify CL tumors in FFPE tumor samples. We tested this profile (TN and low expression of at least two of four epithelial cell-cell adhesion markers; claudin 3, claudin 4, claudin 7 and E-cadherin) in a large cohort of breast tumors with long-term follow up. We have demonstrated that approximately 8% of all invasive breast cancer fall into this unique molecular subtype and the tumor type is characterized by distinguishing morphologic features including high tumor grade, large size and some of the characteristic features of medullary-type cancers. Uniquely we demonstrate that CL tumors have a low incidence of LR following breast conserving therapy (BCT). In addition, CL tumors show an association with known cancer stem cell markers when compared with all other molecular subtypes of breast cancer. While our results are encouraging and suggest that using the IHC panel described, CL tumors can be identified it would be valuable to validate these findings in an independent breast cancer cohort with long-term follow-up,

## Supporting Information

S1 Fig Flow diagram of work. (TIF)

S1 Table Files used for *in silico* analysis. (DOCX)

S1 File Supplementary information for materials and methods. (DOC)

S2 Table Antibodies and conditions of use. (DOCX)

S2 Fig Overall survival (OS) and disease-free survival (DFS) by molecular subtype of the patients belonging to the *in silico* cohort of 1,593 breast cancers. (DOCX)

S3 Fig Box plot illustrating the expression of claudin 7 across the different molecular subtypes of breast cancer. (DOCX)

S4 Fig Composite image of luminal A, luminal B, HER2, Basal and Claudin-low tumors stained with H&E and ALDH1, CD44, CD24 and CD8. (TIF)
