# Supplementary figures and images for: Claudin-Low Breast Cancer; Clinical & Pathological Characteristics
Source: PLoS One. 2017 Jan 3;12(1):e0168669. doi: 10.1371/journal.pone.0168669 (PMC5207440; doi:10.1371/journal.pone.0168669)

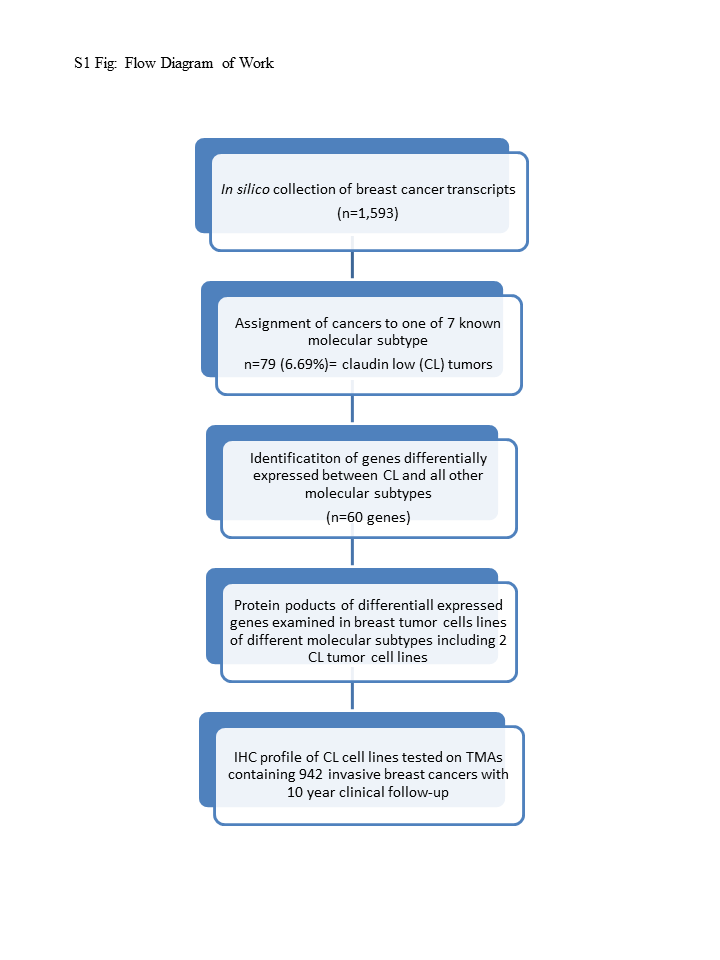

Supplement: S1 Fig — (TIF) [file pone.0168669.s001.tif]

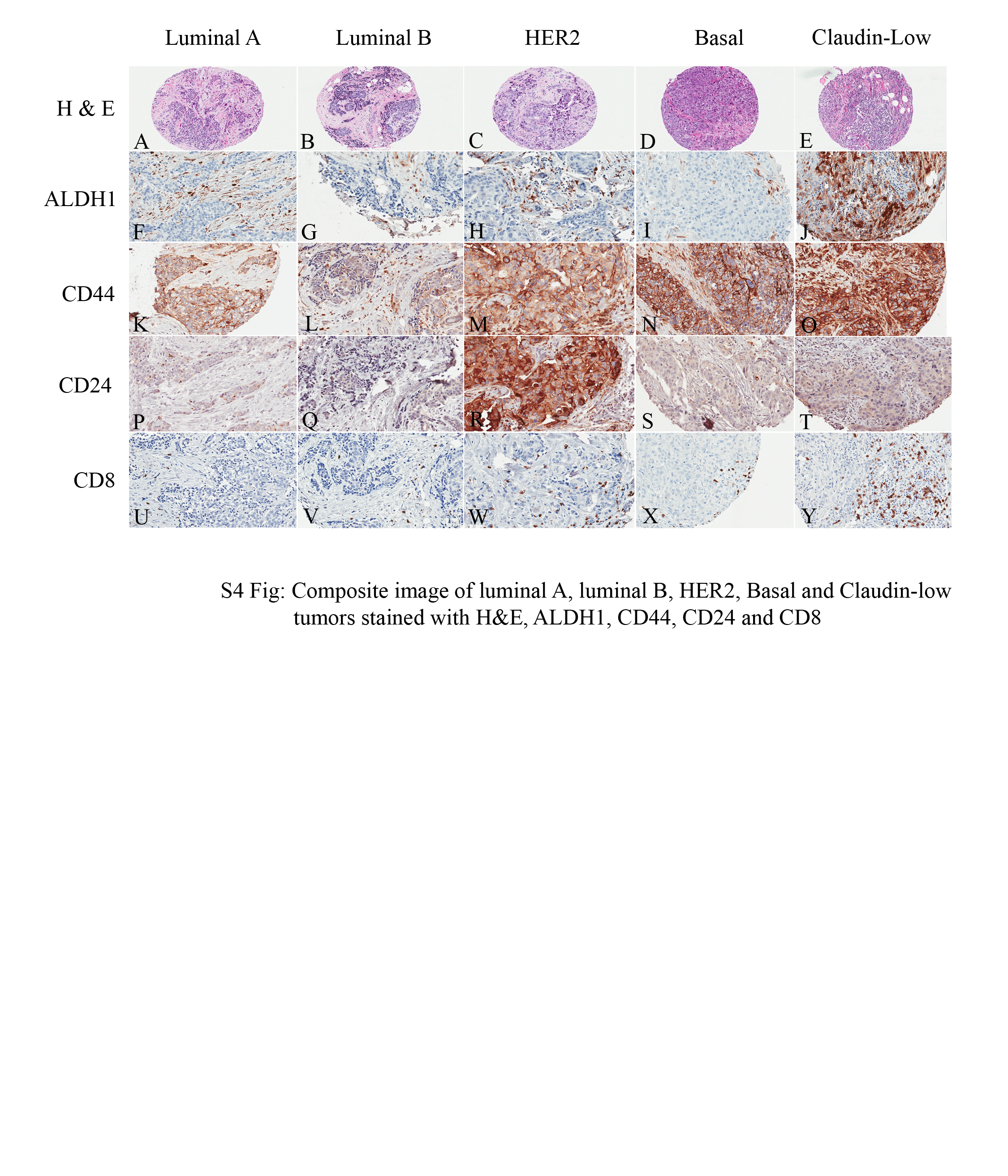

Supplement: S4 Fig — (TIF) [file pone.0168669.s007.tif]
