# Supplementary material for: Claudin-Low Breast Cancer; Clinical & Pathological Characteristics
Source: PLoS One. 2017 Jan 3;12(1):e0168669. doi: 10.1371/journal.pone.0168669 (PMC5207440; doi:10.1371/journal.pone.0168669)
Supplement: S1 Table — (DOCX) [file pone.0168669.s002.docx]

|  | **GSE3494** | **GSE25066** | **GSE17705** | **GSE6532** | **GSE2034** | **GSE7390** | **GSE1456** |
| --- | --- | --- | --- | --- | --- | --- | --- |
| # of patients included in our cohort | 232 | 432 | 174 | 170 | 263 | 184 | 138 |
| wholesection/ macrodissected tumor | After surgery, the viable part of the fresh tumor was cut in two;  one part was immediately frozen in isopentane and stored at -70°C until analysis | a tumor biopsy sample by fine-needle aspiration or core biopsy  prior to any systemic therapy for genomic studies | Fine-needle aspiration | na | frozen tumor samples from tumor bank | frozen samples | frozen  samples |
| ethnicity | na | na | na | na | na | na | na |
| tumour type | na | na | na | na | na | na | na |
| size | 22.3+/- 12.6 | na | na | na | na | 2.2 +/- 0.8 | na |
| lymph node status | 148 (0), 75 (1), 9 (na) | 140 (0), 204 (1), 53 (2), 35 (3) | 102 (0), 68 (1), 4 (na) | na | 263 (0) | 184 (0) | na |
| ER status | 33 (neg), 195 (pos), 4 (na) | 176 (neg), 251 (pos), 2 (0A), 3 (I) | 174 (pos) | 170 (pos) | 69 (neg), 194 (pos) | 61 (neg), 123 (pos) | na |
| PR status | 59 (neg), 173 (pos) | 223 (neg), 203 (pos), 4 (I), 2 (na) | na | na | na | na | na |
| HER2 status | na | 415 (neg), 4 (pos), 3 (I), 10 (na) | na | na | na | na | na |
| Subtypes ** : |  |  |  |  |  |  |  |
| Basal | 20 | 147 | 4 | 15 | 41 | 38 | 15 |
| Claudin-low | 9 | 31 | 7 | 7 | 14 | 6 | 6 |
| HER2 | 18 | 14 | 2 | 10 | 13 | 15 | 9 |
| Luminal A | 56 | 52 | 36 | 26 | 79 | 54 | 37 |
| Luminal B | 32 | 58 | 23 | 25 | 35 | 26 | 17 |
| Normal-like | 40 | 25 | 31 | 25 | 19 | 14 | 25 |
| mApo | 1 | 9 | 2 | 0 | 6 | 2 | 0 |
|  |  |  |  |  |  |  |  |
| Reference | PMID: 16141321 | PMID: 24337596 | PMID: 20697068 | PMID: 17401012 | PMID: 15721472 | PMID: 17545524 | PMID: 16280042 |

**S1 Table: Files used for *in silico* analysis**
