# Supplementary material for: Claudin-Low Breast Cancer; Clinical & Pathological Characteristics
Source: PLoS One. 2017 Jan 3;12(1):e0168669. doi: 10.1371/journal.pone.0168669 (PMC5207440; doi:10.1371/journal.pone.0168669)
Supplement: S1 File — (DOC) [file pone.0168669.s003.doc]

**Supplementary Information**

**Cell line fixation techniques**

Adherent cells were grown on 10cm tissue culture plates until confluent and isolated by scraping. Cell pellets were fixed in 10% formalin for 15 minutes. Fixed cells were mixed with 1% agar and cooled on ice to solidify and fixed in formalin for a further 48hours. Cell pellets were then embedded in paraffin

**Accelerated Hypofractionated Whole Breast Irradiation (AHWBI) Trial Design**

Details of study design have been previously reported {{1545 Whelan 2010;}}. Participants had invasive breast cancer treated by breast conserving surgery and axillary dissection with clear margins of excision and negative axillary nodes. Tumors > 5 cm were excluded. Patients were accrued from nine regional cancer centres in Ontario between the years 1993-1996. Prior to randomization patients were stratified according to age (< 50 years or > 50 years), tumor size (< 2 cm or > 2 cm), systemic adjuvant therapy (tamoxifen, any chemotherapy or no therapy) and treatment centre. Patients were randomly assigned to receive standard whole breast irradiation with a dose of 50 Gy in 25 fractions over a period of 35 days or hypofractionated whole breast irradiation (HWBI) at a dose of 42.5 Gy in 16 fractions over a period of 22 days.

After completion of radiotherapy, patients were seen every six months for 5 years and then yearly. The primary outcome was any LR of invasive cancer in the treated breast. Secondary outcomes were distant (including regional) recurrence, new second cancers (including contralateral breast cancer), overall survival, cosmetic outcome, and toxicity.

**Grading of Tumor Lymphocytic Infiltrate**

None-no lymphoctyes

Minimal-scattered lymphocytes (<10/hpf (40X))

Moderate-easily identified (no large aggregates)

Extensive-large aggregates in >50% of the tumor.
