# Supplementary material for: Claudin-Low Breast Cancer; Clinical & Pathological Characteristics
Source: PLoS One. 2017 Jan 3;12(1):e0168669. doi: 10.1371/journal.pone.0168669 (PMC5207440; doi:10.1371/journal.pone.0168669)
Supplement: S2 Table — (DOCX) [file pone.0168669.s004.docx]

**S2 Table. Antibodies and conditions of use**

| **Antibody against** | **Clone** | **Source** | **Dilution** | **Pretreatment** |
| --- | --- | --- | --- | --- |
| ER | 6F11 | Vector | 1:130 | microwave at 97°C for 20 min in modified Tris (pH 9) |
| PR | PgR1294 | Dako | 1:300 | microwave at 97°C for 20 min in modified Tris (pH 9) |
| HER-2 | A0485 | Dako | 1:400 | microwave at 97°C for 20 min in modified Citrate (pH 6) |
| CK5/6 | VP-C400 | Vector | 1:130 | microwave at 97°C for 20 min in modified Tris (pH 9) |
| EGFR | 31G7 | Invitrogen | 1:35 | microwave at 97°C for 20 min in modified Citrate (pH 6) |
| Ki67 | SP6 | Thermo Scientific | 1:100 | microwave at 97°C for 20 min in modified Citrate (pH 6) |
| E-cadherin | NCH-38 | DAKO | 1:220 | microwave at 97°C for 20 min in modified Tris (pH 9) |
| Claudin 3 | Rabbit Polyclonal | Invitrogen | 1:300 | microwave at 97°C for 20 min in modified Tris (pH6) |
| Claudin 4 | 3E2C1 | Invitrogen | 1:250 | microwave at 97°C for 20 min in modified Tris (pH6) |
| Claudin 7 | 5D10F3 | Invitrogen | 1:250 | microwave at 97°C for 20 min in modified Tris (pH 9) |
| CD44 | DF1485 | Dako | 1:250 | microwave at 97°C for 20 min in modified Tris (pH6) |
| CD24 | SN3b | LAB VISION FISHER | 1:45 | microwave at 97°C for 20 min in modified Tris (pH6) |
| ALDH1A | ALDH | BD Biosciences | 1:1000 | microwave at 97°C for 20 min in modified Tris (pH 9) |
| CD8 | C8/144B | DAKO | 1:100 | microwave at 97°C for 20 min in modified Tris (pH 9) |
|  |  |  |  |  |
