# Supplementary material for: Claudin-Low Breast Cancer; Clinical & Pathological Characteristics
Source: PLoS One. 2017 Jan 3;12(1):e0168669. doi: 10.1371/journal.pone.0168669 (PMC5207440; doi:10.1371/journal.pone.0168669)

**S2 Fig . Overall survival (OS) and disease-free survival (DFS) by molecular subtype of the patients belonging to the *in silico* cohort of 1,593 breast cancers**


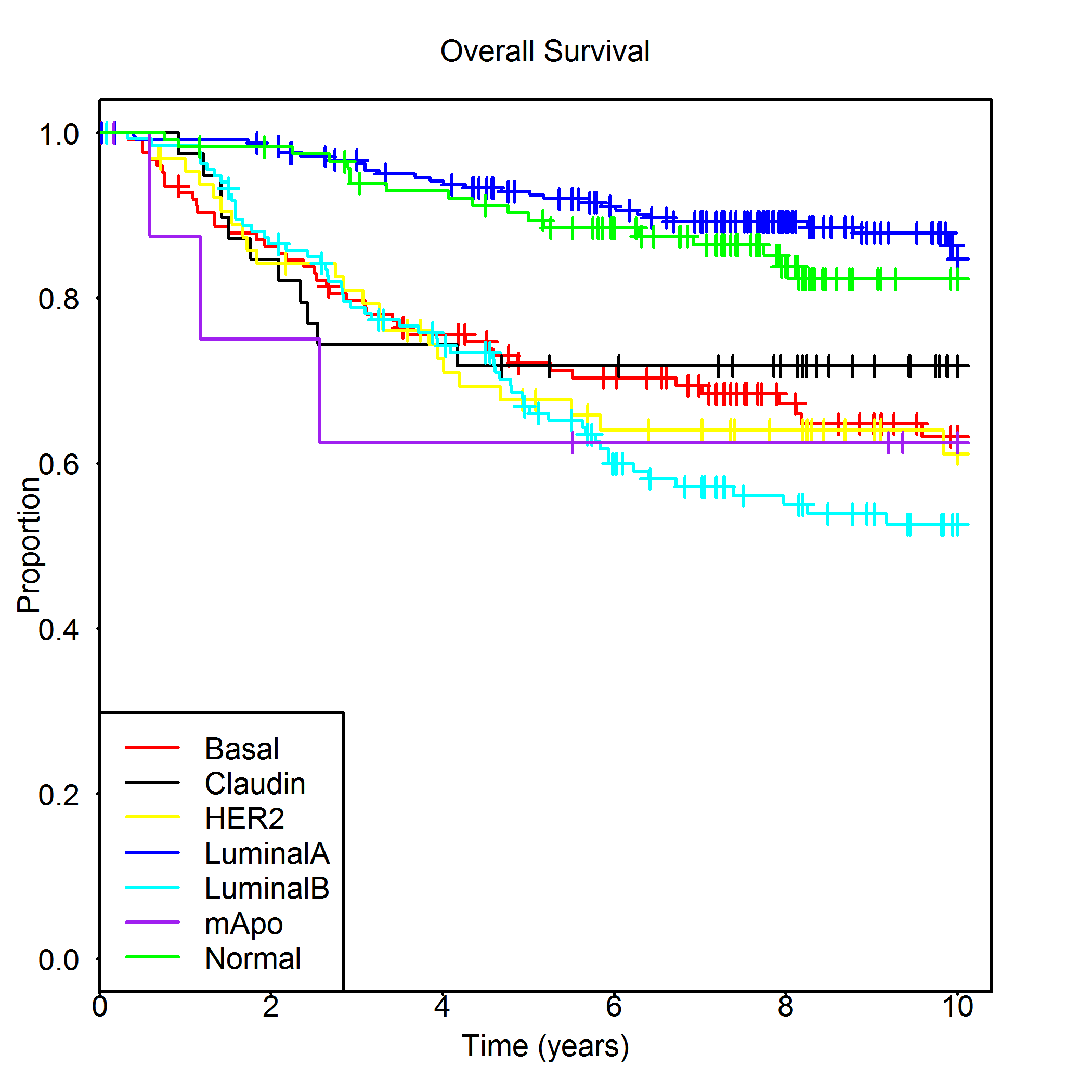


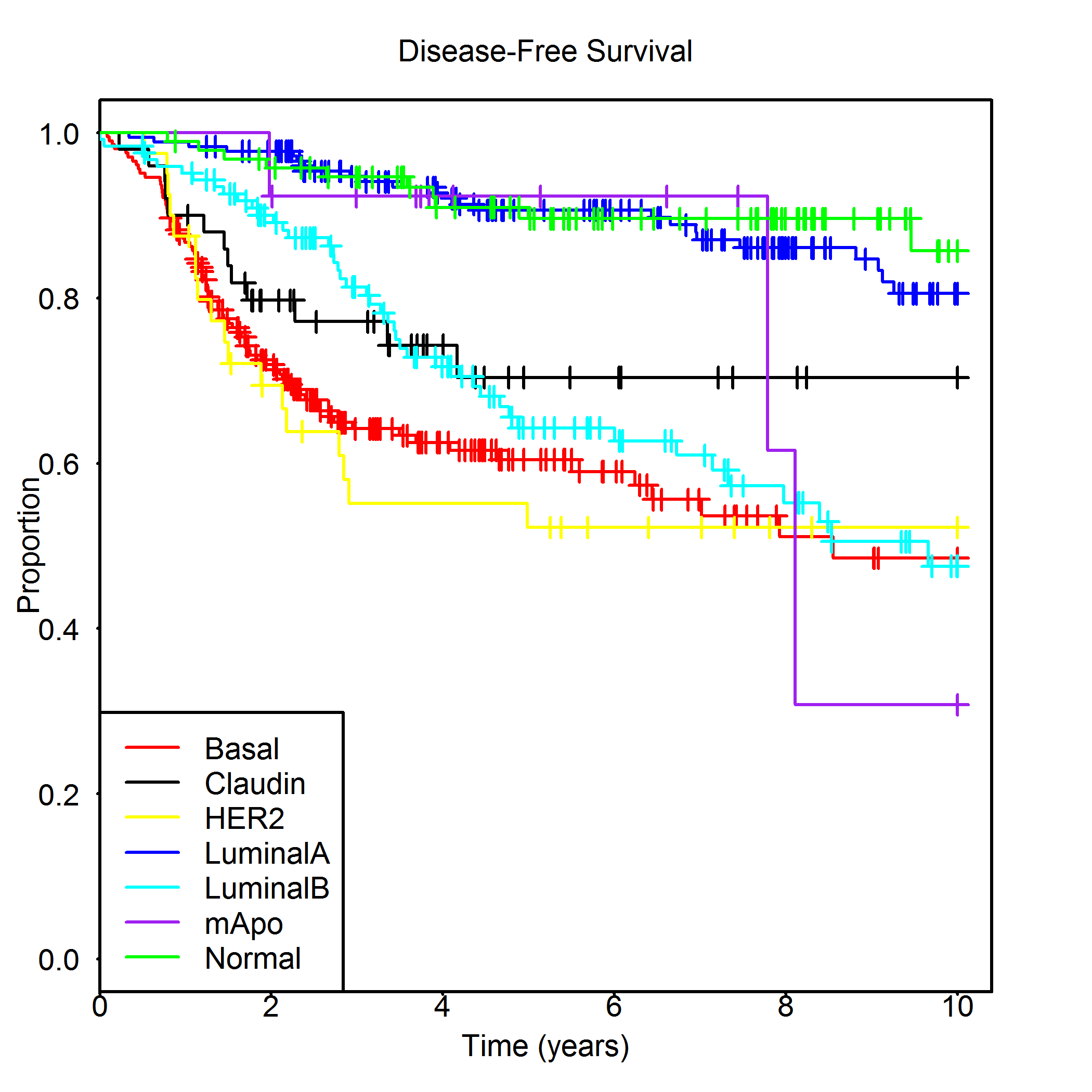

Supplement: S2 Fig — (DOCX) [file pone.0168669.s005.docx]
