# Supplementary material for: Claudin-Low Breast Cancer; Clinical & Pathological Characteristics
Source: PLoS One. 2017 Jan 3;12(1):e0168669. doi: 10.1371/journal.pone.0168669 (PMC5207440; doi:10.1371/journal.pone.0168669)

**S3 Fig . Box Plot illustrating the expression of claudin 7 across the different molecular subtypes of breast cancer**


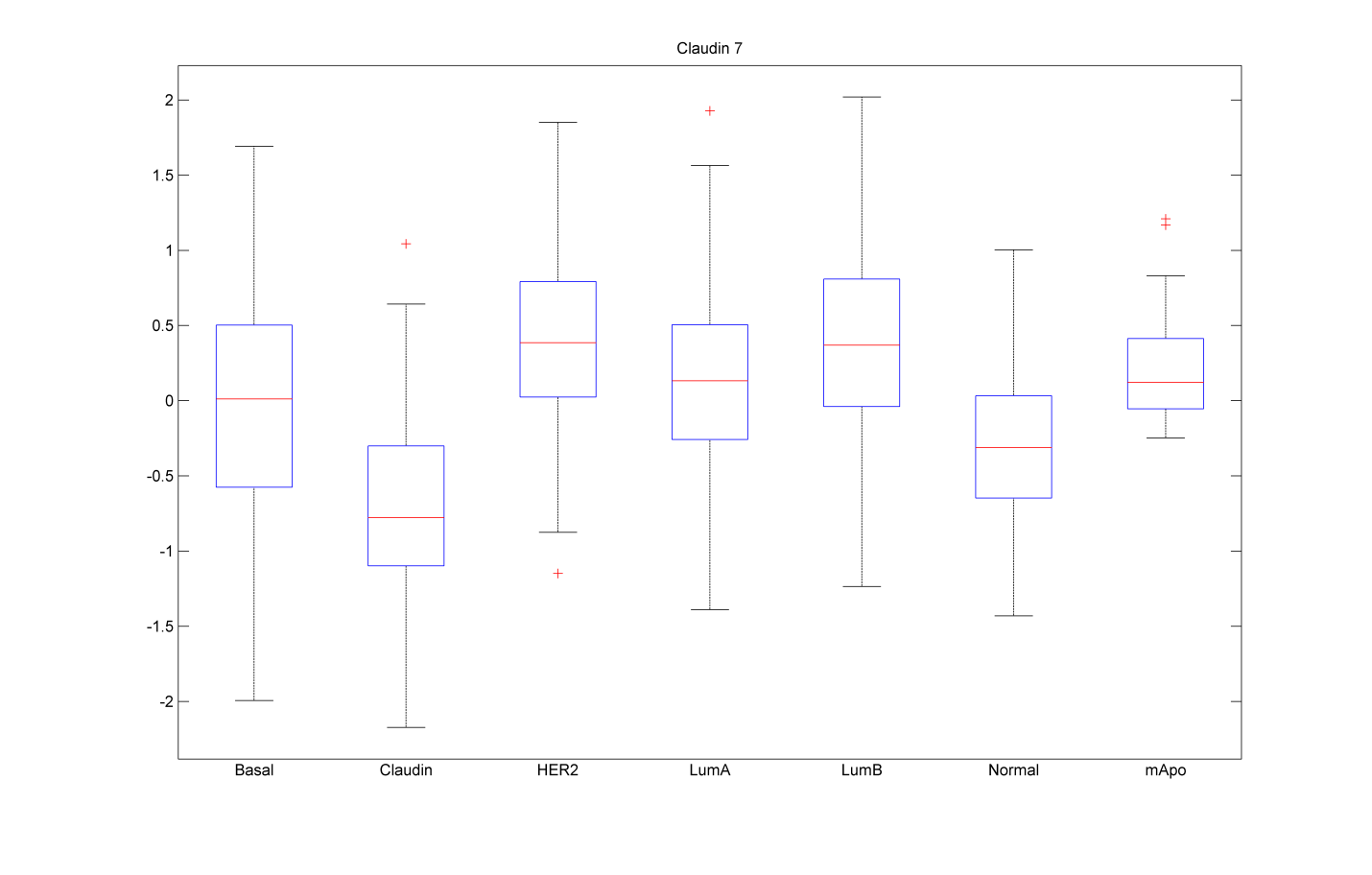

Supplement: S3 Fig — (DOCX) [file pone.0168669.s006.docx]
